# Seed-Borne Bacterial Diversity of Fescue (*Festuca ovina* L.) and Properties Study

**DOI:** 10.3390/microorganisms12020329

**Published:** 2024-02-04

**Authors:** Shaowei Zhu, Jinjing Xie, Jie Yang, Xuan Hou, Linxin He, Zhenfen Zhang

**Affiliations:** Key Laboratory of Grassland Ecosystem, Ministry of Education, Pratacultural College, Gansu Agricultural University, Lanzhou 730070, China; zhusw@st.gsau.edu.cn (S.Z.); xiejj@st.gsau.edu.cn (J.X.); yangjie@st.gsau.edu.cn (J.Y.); houx@st.gsau.edu.cn (X.H.); helx@st.gsau.edu.cn (L.H.)

**Keywords:** *Festuca ovina* L., seed-borne bacteria, motility, biofilm formation, antibiotic resistance

## Abstract

Rich endophytic bacterial communities exist in fescue (*Festuca ovina* L.) and play an important role in fescue growth, cold tolerance, drought tolerance and antibiotic tolerance. To screen for probiotics carried by fescue seeds, seven varieties were collected from three different regions of China for isolation by the milled seed method and analyzed for diversity and motility, biofilm and antibiotic resistance. A total of 91 bacterial isolates were obtained, and based on morphological characteristics, 36 representative dominant strains were selected for 16S rDNA sequencing analysis. The results showed that the 36 bacterial strains belonged to four phyla and nine genera. The Firmicutes was the dominant phylum, and *Bacillus*, *Paenibacillus* and *Pseudomonas* were the dominant genera. Most of the strains had motility (80%) and were biofilm-forming (91.7%). In this study, 15 strains were capable of Indole-3-acetic acid (IAA) production, 24 strains were capable of nitrogen fixation, and some strains possessed amylase and protease activities, suggesting their potential for growth promotion. Determination of the minimum inhibitory concentration (MIC) against the bacteria showed that the strains were not resistant to tetracycline and oxytetracycline. *Pantoea* (QY6, LH4, MS2) and *Curtobacterium* (YY4) showed resistance to five antibiotics (ampicillin, kanamycin, erythromycin, sulfadiazine and rifampicin). Using Pearson correlation analysis, a significant correlation was found between motility and biofilm, and between biofilm and sulfadiazine. In this study, we screened two strains of *Pantoea* (QY6, LH4) with excellent growth-promoting ability as well as broad-spectrum antibiotic resistance. which provided new perspectives for subsequent studies on the strong ecological adaptations of fescue, and mycorrhizal resources for endophytic bacteria and plant interactions.

## 1. Introduction

Excellent forage plant varieties are an important material basis for improving degraded land, adjusting agricultural institutions, establishing grassland agricultural systems and improving the ecological environment [1]. Fescue (*Festuca ovina* L.) is a common forage distributed in the northwest, north and northeast and the Qinghai-Tibet Plateau of China, because of its cold and drought resistance, salinity tolerance, adaptability and good quality. It is often used for lawn restoration and planting of pioneer plants [2]. In addition, fescue also has good feeding value, and in the development of alpine pasture grass construction and ecological management has a very significant economic value. This species is more resistant to adversity than other cool-season turfgrasses, such as perennial ryegrass (*Lolium perenne* L.) or Kentucky bluegrass (*Poa pratensis* L.) [3]. This difference may be attributed to the physiological characteristics of fescue and the role played by microorganisms in different environments. Seeds, as plant propagules, represent an important stage in the plant’s life cycle: they can survive for years in a dormant state and develop into new plants when the conditions are right [4]. Microorganisms are one of the most important organisms that contribute to plant development [5]. In particular, plant-beneficial bacteria can provide many benefits to the host plants, helping them to resist various biotic and abiotic stresses [6]. Endophytic bacteria are a group of microorganisms that are able to colonize and coexist healthily with plants [7]. During seed germination, these endophytic bacteria may interact with surrounding plants to provide benefits to the plants, including phosphorus solubilization, nitrogen fixation, production of growth factors, protection against pathogens, resistance to antibiotics and reduction in stress caused by pollutants [8,9,10], thus significantly affecting soil fertility and plant growth [11]. 

Since their introduction, antibiotics have been used in agriculture, aquaculture and veterinary medicine. Many antibiotic residues are introduced into the environment every year through animal excreta, thus affecting microorganisms in different environments [12]. The plant microbiome is the primary means by which humans are exposed to antibiotic-resistant bacteria and naturally occurring resistance genes in the environment [13]. The phyllosphere (above-ground components) of a plant represents an open habitat dominated by leaves. Antibiotic resistomes associated with phyllospheres can influence human microbiomes by direct consumption and contact, or through the food chain. Plant rhizospheric microbiome (below-ground components) provide nutrient acquisition and resistance to abiotic stresses through contact with the environment [13]. However, antibiotics remaining in the environment can be taken up by plants, mainly through their root systems, thereby affecting nutrient uptake and regeneration under stressful conditions [14]. These antibiotics also induce bacterial gene expression, which can lead to the development of resistance in certain bacteria, posing a threat to animal as well as human health [15,16]. In summary, studies on the potential spread of antibiotic resistance in the environment have focused on the evolution of antibiotic resistance in soil and wastewater, while little attention has been paid to the spread of antibiotic resistance in seeds. Therefore, it is essential to explore the bacterial resistance carried by seeds.

In recent years, the mechanism of bacterial drug resistance has been widely studied as a hotspot. Among these mechanisms, biofilm formation is particularly important. For an antibiotic to be effective, it must cross the biofilm to reach its target [17]. The high adhesion and stable microhabitat of biofilm will prevent the penetration of antibiotics and provide a good barrier for bacteria [18]. Bacterial motility also plays an important role in biofilm formation, and flagellum-driven motility is involved in various aspects of biofilm formation, including biofilm initiation and the recruitment of cells from the motile state, as in the case of *Bacillus cereus*, which is common in both plants and seeds. The mechanism by which motility influences biofilm formation in *B. cereus* has been previously demonstrated [19], and in *B. cereus* ATCC 10987, the majority of mutants exhibiting a film-deficient phenotype also showed impaired motility, suggesting a positive correlation between biofilm formation and motility [20]. In addition, bacterial motility confers the selective advantage of enabling cells to escape harsh conditions and seek more favorable environments [21]. Some rhizospheric or soil-dwelling *Pseudomonas* species act as plant-growth-promoting rhizobacteria (PGPR) migrating to nutrient-rich habitats and avoiding conditions unfavorable to their survival through flagellum-mediated movement [22,23]. This property may also reduce the exposure of bacteria to antimicrobial drugs thereby reducing the damage to the bacteria and allowing them to develop resistance [24,25]. Therefore, the study of bacterial motility and biofilm-forming ability is necessary to study their drug resistance.

Microbial-assisted phytoremediation is a novel and promising concept. To date, a large number of studies have shown that microorganisms can effectively enhance phytoremediation [26]. Many endophytes can also improve host tolerance and help the host to resist different harsh environments. However, there is a paucity of research on antibiotic resistance in fescue seed-culturable bacteria. This study aims to evaluate the cultivable seed-borne bacterial community in seven commercial fescue varieties of different origins. Seed-borne bacteria were isolated, purified and used to define the genus. After isolation, we evaluated the biological properties and functions of these strains, including motility, biofilm formation, antibiotic resistance, auxin production, nitrogen fixation, soluble amylase and protease. These excellent strains can be utilized in ecological restoration and crop improvement programs, which can offer an enormous potential resource of new microbial strains during the synergistic plant-microbe interaction under antibiotic stress.

## 2. Materials and Methods

### 2.1. Experimental Materials 

A total of seven seed lots of commercial varieties of fescue were collected from different regions and stored at −4 °C in the Laboratory of Forage Germplasm Resources, Pratacultural College, Gansu Agricultural University (Lanzhou, China). Their related information is shown in Table 1.

### 2.2. Isolation and Identification of Seed-Borne Bacteria from Fescue Seeds

One gram of seeds (approximately 520 seeds) were weighed and placed into a sterile beaker for surface disinfection (75% ethanol for 2 min, 3% sodium hypochlorite for 6 min). Subsequently, they were washed thrice with sterile distilled water. After surface sterilization, the seeds were moved to a mortar with 10 mL of sterile water and ground within a biosafety cabinet. Following thorough grinding, the samples stood undisturbed for 10 min. The experiment involved six dilution gradients (10^0^–10^−5^), with 200 μL of supernatant aspirated and uniformly spread on the Tryptose Soya Agar solid medium (TSA: tryptone 15 g/L; peptone 5 g/L; NaCl 5 g/L; agar 15 g/L and 1 L dis. H_2_O, adjusted to pH 7). Sterile distilled water was used as the control group, and the experiment was repeated three times. Subsequently, the plates were incubated at 28 °C in a light incubator (SPX-250-GB, Shanghai Yuejin Medical Equipment Co., Shanghai, China) for 48–72 h. Each of those morphologically differentiable bacterial colonies was repeatedly streaked on plates of Luria–Bertani medium (LB: tryptone 10 g/L, yeast extract 5 g/L and NaCl 5 g/L; pH 7.2) for purification until a complete isolation was achieved, at which point the colony was cryopreserved at −80 °C in glycerol stocks (LB media plus 25% glycerol) [27,28].

The strain was revived on TSA solid medium. Genomic DNA was then extracted using the Bacterial Genomic DNA Extraction Kit (TIANGEN, Beijing, China). Universal primers 27F (5′-AGAGTTTGATCCTGGCTCAG-3′) and 1492R (5′CTACGGCTACCTTGTTACGA-3′) were selected for PCR amplification [29]. Subsequently, the PCR products were sent to Shanghai Parsonage Biotechnology Co. (Shanghai, China) for sequencing. The quality of DNA and PCR products was assessed through agarose gel electrophoresis and DNA nanodrop. The obtained sequences were then subjected to a BLAST search on the NCBI database, recording the closest matches (http://www.ncbi.nlm.nih.gov/blast, accessed on 27 November 2023). The sequences, along with their closest matches, were submitted to the NCBI to obtain accession numbers for the respective isolates. The software MEGA 11 was employed for sequence analysis and phylogenetic tree construction.

### 2.3. Determination of Biological Properties and Functions

#### 2.3.1. Determination of Motility of Seed-Borne Bacteria

The swimming motility of bacteria was determined by semi-solid puncture method. Single colonies of bacteria were picked with sterile toothpicks and incubated on solid medium (0.3% agar) at 28 °C for 24 h. The diameter of the turbid area formed by the migration of bacteria from the inoculation point was observed and measured, and the test results were recorded [30].

#### 2.3.2. Biofilm-Forming Capacity 

The isolated Gram-positive strains were suspended in Trypticase Soy Broth (TSB: tryptone 15 g/L; peptone 5 g/L; NaCl 5 g/L; and 1 L dis. H_2_O, adjusted to pH 7) supplemented with additional 1% glucose, and the Gram-negative strains were suspended in LB for incubation at 37 °C for 16–18 h. After incubation, the bacterial suspension was adjusted to a 0.5 McFarland standard (OD625 nm, 0.08–0.13). The inoculation was diluted with sterile TSB or LB at a ratio of 1:100. Then, 150 μL of the bacterial suspension was transferred to sterile 96-well plates using a multichannel pipette, each plate containing 11 strains, and the negative control was uninoculated TSB or LB, each containing 4 wells; Each experiment was repeated three times. Inoculated plates were incubated in an incubator at 37 °C for 48 h. After 48 h, the liquid was poured out and all wells were emptied. Each well was rinsed with sterile 250 μL 0.9% normal saline three times, followed by the addition of 150 μL of 0.1% crystal violet staining solution to each well for staining. After being left for 15 min, the color was gently shaken off, and each well was rinsed three times with 250 μL of distilled water and dried. Finally, 150 μL of 96% ethanol was added to each well. The optical density (OD) of the wells was then measured at 570 nm using a UV-9000 S UV-visible spectrophotometer (Shanghai Metash Instruments Co., Shanghai, China). The biofilm formation ability could be determined as follows: OD ≤ ODc for no biofilm formation, OD < OD ≤ 2ODc for weak biofilm formation, 2ODc < OD ≤ 4ODc for medium biofilm formation and OD > 4ODc for strong biofilm formation [31,32].

#### 2.3.3. Determination of Minimum Inhibitory Concentration (MIC)

The MIC (mg/L) of seven antibiotics (ampicillin, kanamycin, erythromycin, oxytetracycline, tetracycline, sulfadiazine and rifampicin) was determined by agar dilution method. TSA solid medium containing antibiotics at concentrations ranging from 1–512 mg/L were prepared by the twofold dilution method. Isolated bacteria were inoculated into TSB and incubated for 24 h in the dark in an incubator (SPX-250-GB, Shanghai, China) at 28 ± 1 °C. After incubation, the bacterial suspension with absorbance of 0.08–0.13 at OD625 nm was obtained by dilution. After that, 2 µL of the bacterial suspension was inoculated on antibiotic-containing and antibiotic-free TSA, and repeated three times. The strains cultured on TSA without antibiotics were used as controls. After incubation at 37 °C for 48 h, the MIC value was recorded as the minimum inhibitory concentration of the antibiotic [33,34]. 

#### 2.3.4. Measurement of Indole-3-Acetic Acid (IAA) 

The colorimetric method of Gordon and Weber (1951) was followed to assess the production of IAA by bacteria in LB broth cultures using Salkowski reagent (0.5 M FeCl_3_, 1 mL; dis. H_2_O, 50 mL; H_2_SO_4_, 30 mL). All bacteria were incubated in LB medium for 48 h at 28 °C, then 2 mL was centrifuged and the supernatant was collected for testing. One milliliter of the supernatant was mixed with 2 mL of freshly prepared Salkowski reagent and incubated for 30 min before recording the absorbance at 530 nm. The test was repeated three times. The concentration of IAA produced per mL of bacterial culture was estimated by comparing the absorbance of the standard curve. To plot the standard curve, different concentrations of IAA (0.0, 5.0, 10.0, 20.0, 30.0, 40.0, 50.0, 60.0 μg/mL) were prepared in LB broth medium and then 1 mL of each concentration was mixed with 2 mL of Salkowski reagent. The absorbance was recorded at 530 nm after incubation for 30 min. The absorbance was plotted against the concentration of IAA to obtain a standard curve [29,35].

#### 2.3.5. Nitrogen Fixation and Extracellular Enzyme Activity 

For the nitrogen fixation test, all bacterial cultures were inoculated with Ashby’s Medium (KH_2_PO_4_ 0.2 g, NaCl 0.2 g, MgSO_4_ 0.2 g, CaCO_3_ 5 g, K_2_SO_4_ 0.1 g, glucose 10 g, agar 15 g) and placed in an incubator at 28 °C, and growth was observed after the 7th day of incubation [36]. Amylolytic activity were assessed by growing the endophytic bacterial isolates on TSA agar medium supplemented with 1% soluble starch. After incubation, the plates were filled with iodine solution (1% iodine in 2% potassium iodide solution) and the clear circle around the colonies was measured as a result of the assay [37]. The overnight cultured suspensions were inoculated onto nutrient agar plates (pH 10) containing 10% milk (*v*/*v*) [28] and incubated for 48 h at 28 ± 1 °C for qualitative screening of protease activity. After incubation, the diameter of the clear zone around the colonies was recorded to indicate the extent of protease activity.

### 2.4. Statistical Analysis

All data indicators are expressed as mean ± standard error of three replicates. Data were collated using Excel 2021, analyzed by one-way ANOVA using SPSS 23.0, and separated by means using Duncan’s test; *p* < 0.05 indicates significance. The heatmap was carried out using the Chiplot platform (https://www.chiplot.online/, accessed on 15 October 2023).

## 3. Results

### 3.1. Fescue Seeds from Different Varieties as Natural Carriers of Taxonomically Diverse Culturable Seed-Borne Bacteria

**Figure 1:**
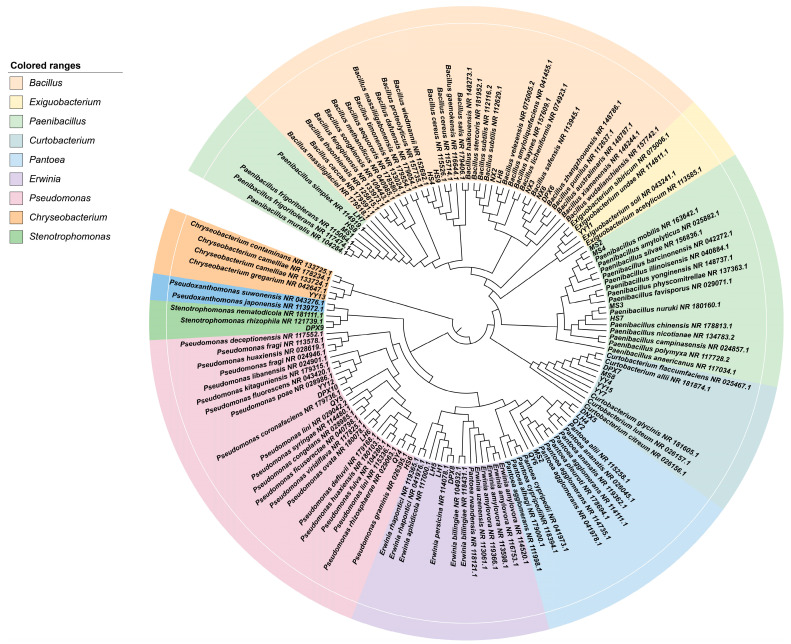
The phylogenetic tree of isolated endophytic bacterial strains was constructed using the neighbor-joining method by MEGA 11 software.

A total of 91 strains of bacterial isolates were isolated from 7 different varieties of fescue seeds, and there were significant differences in the number of strains isolated from each variety (*p* < 0.05). Based on the morphological features encompassing colony morphology, size, gloss and pigment production, 36 representative bacterial isolates were chosen for 16S rDNA identification. Among the 36 representative strains, 7 strains were identified as *Bacillus*, 7 strains as *Paenibacillus*, and 1 strain as *Exiguobacterium*, all falling within the Firmicutes. Furthermore, there were six strains of *Pseudomonas*, five strains of *Pantoea*, three strains of *Erwinia* and one strain of *Stenotrophomonas*, classified under the Proteobacteria. Within this group, five strains of *Curtobacterium* were assigned to Actinobacteria, and one strain of *Chryseobacterium* was associated with Bacteroidetes. Following this, Gram staining was conducted on the selected strains, revealing 15 Gram-positive strains (G+), constituting 41.77% of the total strains. The remaining 21 strains were Gram-negative (G−), representing 58.23%. (Figure 1 and Table 2).

### 3.2. Community Structure and Abundance Analysis of Seed-Borne Bacteria

**Figure 2:**
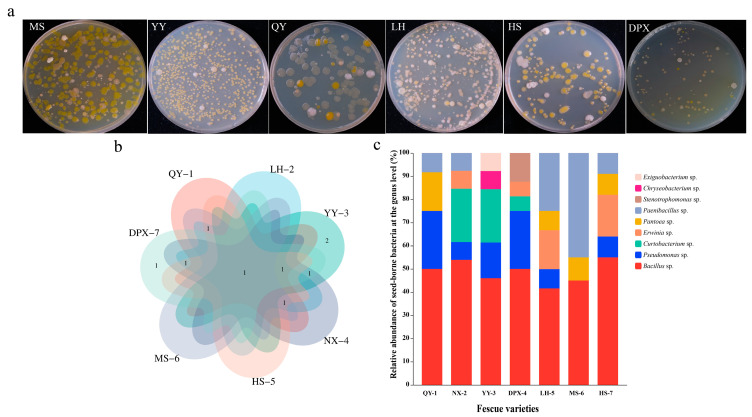
Seed-borne bacterial diversity. (**a**) Diversity of bacteria on plates (10^−2^). MS: *F. rubra* ‘dream-God’; YY: *F. arundinacea* ‘bharal’; QY: *F. sinensis* (Qinghai); LH: *F. arundinacea* ‘road-Fire’; HS: *F. kryloviana*; DPX: *F. arundinace* ‘horizon’ (**b**) Venn analysis of diversity. (**c**) Relative abundance (%) of seed-borne bacteria between varieties at the genus level.

We selected plates with colony counts between 30–300 colony forming units (CFU) and no spreading colony growth for the counting of colonies, and the average of three replicates was used for each dilution. The number of bacteria isolated from the experiment was calculated to be 10^4^–10^6^ CFU/g. Strains belonging to *Bacillus*, *Pseudomonas*, *Paenibacillus*, *Curtobacterium*, and *Pantoea* were identified in the seeds of seven distinct varieties. In addition, the bacteria obtained from diverse varieties exhibited a range of diversity, With the exception of ‘Qinghai’ *F. sinensis* and ‘dream-God’ *F. rubra.* the seed-borne bacteria of the remaining five varieties of fescue were relatively abundant at the genus level, containing five genera (Figure 2c). The common and endemic genera of seed-borne bacteria of different varieties were presented by Venn diagrams, among which, ‘bharal’ *F. arundinacea* had two endemic genera, *Chryseobacterium* and *Exiguobacterium*, and ‘horizon’ *F. arundinacea* had one endemic genus, *Stenotrophomonas* (Figure 2b). It is worth noting that *Bacillus* was the predominant genus in all varieties. Our study showed that different numbers of culturable bacteria were present in different fescue seeds, which in turn included diversified bacteria of different genera. The duration of different varieties of seeds stored at 4 °C may be the source of bacterial species heterogeneity in different varieties. Other sources of heterogeneity may come from differences in seed genotypes themselves, or from differences in plant and soil microbiomes associated with each batch of seed production.

### 3.3. IAA Production, Nitrogen Fixation Capacity, Soluble Amylase and Protease Activity

Out of 36 bacteria evaluated for IAA production, HS9 produced the highest levels of auxin (40.88 ± 0.709 μg/mL), followed by QY6 (40.10 ± 0.233 μg/mL) and LH4 (39.48 ± 0.337 μg/mL). Twenty-four bacterial strains exhibited nitrogen fixation activity, 8 strains of bacteria exhibited amylase activity, 11 strains exhibited protease activity, and strains QY6, NX5, HS9, YY12 and DPX7 possessed both amylase and protease activities (Table 3).

### 3.4. Motility of Seed-Borne Bacteria

**Figure 3:**
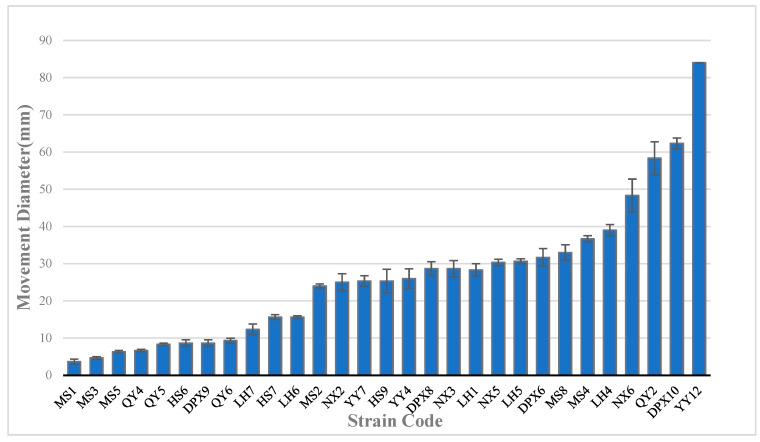
Motility of seed-borne bacteria, with the size of the motility diameter representing strength.

Swimming bacteria can monitor changes in environmental conditions during their movement and adjust their swimming patterns accordingly in order to swim to their preferred environment. In this experiment, motility was assessed based on bacterial performance on semi-solid agar medium. The findings revealed that out of the 36 bacterial strains, 29 exhibited varying degrees of motility, while 7 strains displayed no motility (Figure 3). Among them, YY12, with the strongest exercise ability, showed strong movement on agar plates. After 24 h of culture, it formed a swollen circular colony, which was significantly stronger than other bacteria (*p* < 0.01). DPX10 and QY2 exhibited the second highest motility, with moving diameters of 62 mm and 58 mm, respectively. The first two bacterial strains belonged to *Pseudomonas*, and QY2 belonged to *Pantoea*. Furthermore, aside from seven non-motile strains, the *Bacillus* strain (MS1), demonstrated the poorest motility with a diameter of 3.6 mm, significantly lower than other bacteria (*p* < 0.05).

### 3.5. Biofilm-Forming Ability

**Figure 4:**
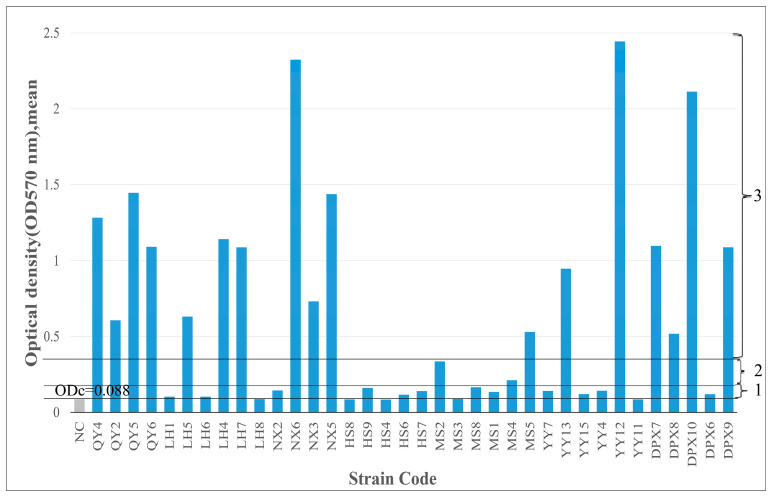
Biofilm-forming ability of 36 strains of seed-borne bacteria on 96-well plates. TSB containing 1% glucose was used as a negative control (NC). The cut-off value (ODc) and the level of biofilm production capacity are marked with horizontal lines: below ODc indicates no biofilm production, 1—weak biofilm-producing bacteria; 2—moderate biofilm-producing bacteria; 3—strongly connected biofilm-producing bacteria.

Biofilm detection was performed on 36 strains of isolated bacteria. Among them, 12 strains (33.3%) were moderate or strong biofilm producers; 21 strains (58.4%) were weak biofilm producers; and 3 strains (8.3%) were biofilm nonproducers. *Bacillus* strains were predominantly biofilm producers. Although *Bacillus* bacteria were present in various fescue seeds varieties, their biofilm-forming capabilities differed significantly (*p* < 0.05), possibly attributed to the seed production environment and inherent bacterial characteristics (Figure 4).

### 3.6. Antibiotic Resistance of Seed-Borne Bacteria

**Figure 5:**
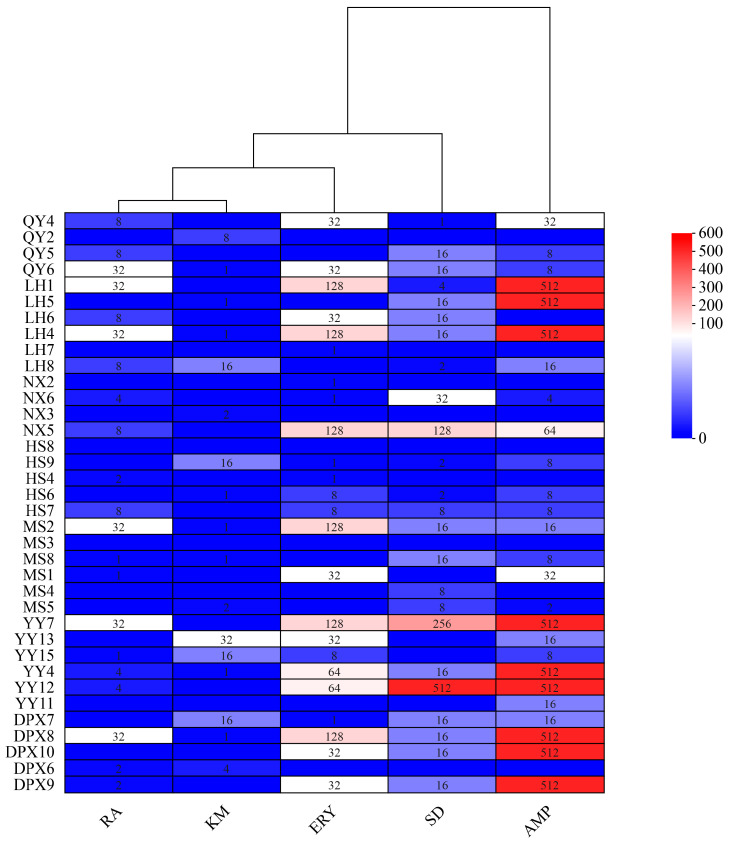
Heatmap of the distribution of MIC (mg/L) of 36 strains of fescue seed-borne bacterial strains. SD: Sulfadiazine; AMP: Ampicillin; KM: Kanamycin; ERY: Erythromycin; RA: Rifampicin.

The susceptibility of 36 strains of seed-borne bacteria to 7 antibiotics was determined by agar dilution method, and the distribution of MIC of the test strains is shown in Figure 5. No strains exhibited resistance to tetracycline and oxytetracycline, while 24 strains displayed varying degrees of resistance to sulfadiazine, 3 strains demonstrated elevated levels of resistance to sulfadiazine (128, 256, and 512 mg/L, respectively); Within the 36 strains of bacteria, 20 exhibited varying degrees of resistance to ampicillin, with 9 strains displaying high-level resistance, reaching a MIC value of 512 mg/L. Additionally, 17 strains showed resistance to kanamycin, with the maximum MIC value being 32 mg/L. Among the 24 strains resistant to erythromycin, 6 strains displayed resistance, each with a MIC value of 128 mg/L. Furthermore, 21 strains demonstrated resistance to rifampicin, with the maximum MIC value being 32 mg/L. Notably, four strains (QY6, LH4, MS2, YY4) exhibited resistance to five antibiotics, while two strains (MS3, HS8) displayed no resistance to all antibiotics.

### 3.7. Correlation Analysis

**Figure 6:**
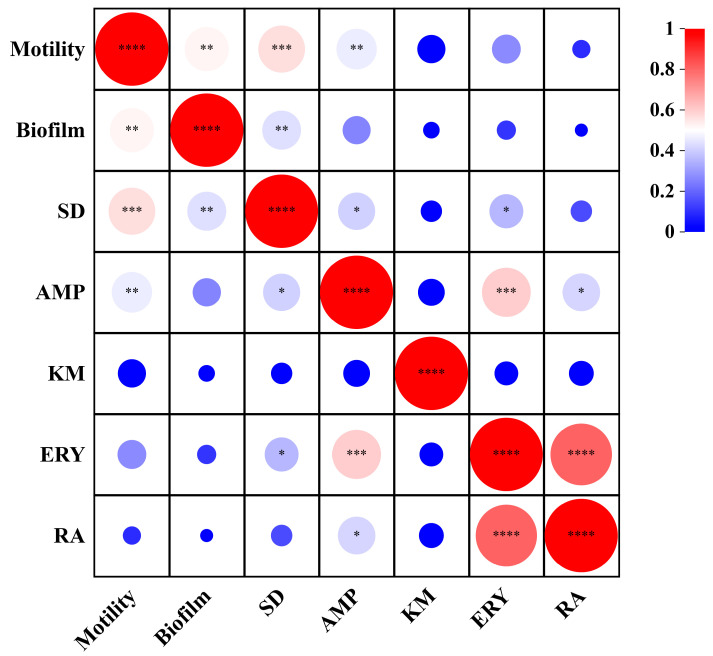
Heatmap of correlation between motility, biofilm and antibiotic resistance. *, *p* < 0.05; **, *p* < 0.01; ***, *p* < 0.001, ****, *p* < 0.0001.

To explore the relationship between bacterial biological properties, the correlation among motility, biofilm and antibiotics resistance indexes was analyzed using Pearson correlation analysis (Figure 6). The results revealed that most of the bacteria isolated in this study had motility, and there was a significant positive correlation between motility and biofilm formation ability (r = 0.52, *p* < 0.01), indicating that the strength of motility was closely related to the formation and change of biofilm. There was a significant positive correlation between exercise and sulfadiazine (r = 0.56, *p* < 0.01) and ampicillin (r = 0.46, *p* < 0.01). Furthermore, the correlation analysis of biofilm and antibiotics resistance showed that the biofilm was positively correlated only with sulfadiazine (r = 0.43, *p* < 0.01) and negatively correlated with all other antibiotics. Interestingly, our study obtained where certain bacteria exhibited robust motility but showed weak biofilm formation. We hypothesize that this phenomenon may be attributed to inherent bacterial properties.

## 4. Discussion

In this study, we isolated and characterized culturable bacteria from different varieties of fescue seeds and found that the bacterial varieties and content varied among different fescue seeds, confirming the existence of a significant diversity of fescue seed-borne bacteria. In 7 seed samples, we identified 36 strains of seed-borne bacteria belonging to 4 phyla and 9 genera, namely, Firmicutes, Proteobacteria, Actinobacteria and Bacteroidetes. Firmicutes was the main phylum, and Proteobacteria and Actinobacteria were the second-most common. This is in agreement with previous studies. The 131 endophytic bacterial genera reported in previous studies present in 25 different plants or seeds were derived mainly from Firmicutes, Proteobacteria, Actinobacteria and Bacteroidetes, including bean (*Phaseolus vulgaris*), rice (*Oryza sativa*), maize (*Zea mays*), etc. [38,39,40,41]. Further analyzed at the genus level, the bacteria isolated were from nine genera, including *Bacillus*, *Paenibacillus*, *Exiguobacterium*, *Pseudomonas*, *Pantoea*, *Erwinia*, *Stenotrophomonas*, *Curtobacterium* and *Chryseobacterium*. Among these bacteria, *Bacillus* is the most prominent genus. The presence of *Bacillus* as a dominant endophyte in alfalfa seeds and rice has also been reported in existing studies, which is consistent with our study [28,29]. As a seed endophyte, *Bacillus* plays an important role in plant growth and development, including seed germination and seedling growth; in addition, it has been shown that the endophytic spores produced by *Bacillus* facilitate seed storage and protection. Therefore, *Bacillus* may play an important role as a core bacterium present in seeds at different developmental stages of the plant [42]. The coexistence of microorganisms with plants contributes to plant adaptation to the environment, and in turn, the plant microbial community responds to the environment and the host to the benefit of the plant [43]. Seeds store the propagation of plants from one generation to another, and seed evolution may be related to a wide range of microorganisms that affect plant growth [44,45]. Differences in the community structure of microorganisms are influenced by many factors. Different environmental conditions affect the reproduction and spread of various microorganisms, and different species carry different types and numbers of microorganisms; even the same seed may carry different microorganisms under different growth conditions [46]. Environmental factors have different effects on the abundance and diversity of seed-borne microorganisms [43]. In this study, we collected fescue seeds from three regions and the isolation results showed diversity. Both shared and exclusive genera were found in the same species from the same region and in the same species from different regions, which just shows that the growing environment may be an important factor for seeds to carry different microorganisms. Unfortunately, we have only studied culturable bacteria in seeds, which represent only a fraction of the seed bacterial community, so we are not able to fully characterize the diversity of the community of seed-borne bacteria. Secondly, microorganisms are selective for culture media, and the media we used may not be able to meet the needs of all microorganisms for growth, which is a limitation.

There is a lot of interest in developing bacteria for biofertilizer and biocontrol applications [47]. Indole-3-acetic acid (IAA) production and nitrogen fixation have been recognized as two important abilities to promote plant growth, especially IAA production. IAA is a major plant growth auxin involved in a variety of plant physiological processes. IAA produced by endophytic bacteria enhances plant root biomass and increases plant yield [48,49]. In this study, it was found that 41.7% of the bacteria were able to produce IAA, especially strains QY6 and HS9, with IAA-producing capacity exceeding 40 μg/mL; most of the bacteria had nitrogen-fixing capacity and 11 strains possessed both capacities. In addition, we found that most of these IAA-producing and nitrogen-fixing bacteria were *Bacillus*. Gagne-Bourgue and colleagues found that inoculating plants with IAA-producing *Bacillus* improved plant growth compared to uninoculated controls (Gagne-Bourgue, 2013). Another study reported that IAA-producing endophytic bacteria isolated from terrestrial orchids significantly increased the length and number of roots, suggesting that bacterial IAA plays an important role in the development of plant roots [50]. In the study of bacterial enzyme activities, we observed that 30% of bacteria had protease activity and 22% had amylase activity.. Endophytic bacteria producing hydrolytic enzyme are involved in the indirect promotion of plant growth, and these amylase- and protease-producing bacteria increase the utilization of endospermic starch in germination and seedling formation. Another study also showed that the production of hydrolytic enzymes by bacterial endophytes helped to improve seedling growth and promote plant development [37]. We can screen these endophytes for specific properties such as biological nitrogen fixation, plant growth promotion, biocontrol and phytoremediation. These properties are particularly relevant for the application of perennial crops, which are often grown under low-input conditions, on low-quality land, and are therefore tolerant to a wide range of abiotic stresses [8].

Properties of bacteria are important for plant colonization as well as resistance to stress, including motility and biofilms. Swimming motility, together with chemotaxis, provides bacteria with the ability to enter the host on their own and is considered an important way to enhance growth and survival [51,52]. For many bacteria, biofilms are a key adaptive trait with selective advantages that allow them to survive in harsh conditions and seek out favorable living environments [21]. On the other hand, biofilms are the primary mode of life for microorganisms, providing them with a protective environment that allows them to perform important functions [53]. In our study, most of the strains isolated in this study were motile (80%) and biofilm-forming (91.7%), and these properties may be the key to help them colonize the seeds. Microbial-plant interactions are prevalent in ecosystems, where beneficial microorganisms are passed directly to the next generation by vertical transmission. In contrast, acquiring microorganisms from the environment requires that motile microorganisms colonize symbiotic partners through specific signaling molecules produced by the host [51]. *Bacillus cereus* and *Bacillus thuringiensis*, which are found in a variety of plants and seeds, produce spores to sustain themselves by forming biofilms in the rhizosphere and foliar surfaces, thus colonizing germinating plants [21]. In addition, there seems to be a close relationship between bacterial motility and biofilm formation. Our study found a significant positive correlation between motility and biofilms (*p* < 0.01). In a study to construct *Bacillus cereus* mutants to reveal genes required for biofilm formation, it was found that the majority of mutants exhibiting biofilm defects also showed impaired motility, and that there was a positive correlation between biofilm formation and motility in constructed models of thin films [20], suggesting that motility is an important factor in biofilm formation, and this relationship was also found in our study. In summary, motility is a widespread trait, and swimming is the most direct apparent behavior of prokaryotes. Motility not only helps bacteria to escape from harsh environments, but also helps them to form biofilms faster and protect themselves from invasion, so that they can search for better and more stable environments.

Antibiotics can negatively affect plant seed germination, but the effects vary depending on the plant species and the antibiotic used in the test [54]. Whereas for bacteria in seeds, antibiotic susceptibility depends to a large extent on the bacteria carrying antibiotic resistance genes [10]. Secondly, bacteria can exert resistance through their ability to form biofilms (e.g., the non-specific barrier provided by the outer membrane of Gram-negative bacteria), which also means that bacteria attached to surfaces and growing as biofilms can resist antibiotic damage [55]. Our study revealed different responses of various bacterial strains to seven antibiotics. All strains were not resistant to tetracycline and oxytetracycline; 94% of the strains had varying degrees of resistance to different antibiotics; and the strains (QY6, LH4, MS2, and YY4, respectively) were resistant to five antibiotics. Notably, we found differences in the response to antibiotics among different species of the same genus, which we hypothesized may be due to differences in biofilm formation ability among strains, which can enhance bacterial resistance thereby helping bacteria to survive under different environmental conditions. However, our study only found a significant correlation between biofilm-forming ability and sulfadiazine (*p* < 0.01), and negatively correlated with other antibiotics. This may be due to the fact that the mechanism of bacterial resistance to antibiotics is complex and includes a series of gene-level determinants such as antibiotic-substrate component interactions, reduced growth rates, and other factors that specifically mediate resistance in the biofilm growth pattern [55].

Briefly, there have been no studies on culturable bacteria in the seed-borne of fescue, and it is the first time that the properties and antibiotic resistance of bacteria endogenous to fescue seeds have been investigated, based on which we isolated and identified bacteria carried in different varieties of fescue seeds, and then analyzed their biological properties and biological functions. This will help us screen strains with antibiotic resistance and provide strain resources for subsequent exploration of plant-microbe interactions under antibiotic stress. It also provides a theoretical basis for ameliorating antibiotic-contaminated land.

## 5. Conclusions

We isolated and characterized 36 strains of seed-borne bacteria from the seeds of seven different varieties of fescue, and the results showed that the 36 strains of seed-borne bacteria belonged to 4 phyla and 9 genera, of which the Firmicutes was the dominant phylum, and the dominant genus was *Bacillus*. In our study on the functions of seed-borne bacteria, we found five strains of bacteria (QY6, NX5, HS9, YY12, DPX7) with excellent growth-promoting properties, as well as IAA-producing ability, nitrogen-fixing ability and amylase activity and protease activity. They can be applied to plants as biotrophic bacteria. In addition, this study explored the antibiotic resistance, motility and biofilm formation ability of seed-borne bacteria, we found that most of the strains had motility (80%) and were biofilm-forming (91.7%), and the strains were not resistant to tetracycline and oxytetracycline. *Pantoea* (QY6, LH4, MS2) and *Curtobacterium* (YY4) showed resistance to five antibiotics (ampicillin, kanamycin, erythromycin, sulfadiazine and rifampicin); all of these properties are related to the survival and adaptive capacity of the bacteria. Using Pearson correlation analysis, a significant correlation was found between motility and biofilm; and between biofilm and sulfadiazine. Based on the results of the study of the biological functions and properties of bacteria, we screened two strains of *Pantoea* (QY6, LH4) with excellent growth-promoting ability as well as broad-spectrum antibiotic resistance. which provided new perspectives for subsequent studies on the strong ecological adaptations of fescue, and mycorrhizal resources for endophytic bacteria and plant interactions.

## Figures and Tables

**Table 1:** Origin and information of seed samples tested.

Variety	Production Region	Production Year	Longitude (E)	Latitude (N)	Seed Provider
*F. arundinacea* ‘horizon’	Beijing, China	2022	116°23′51″	39°54′24″	Beijing Clover Company Beijing, China
*F. arundinacea* ‘bharal’	Beijing, China	2022	116°23′51″	39°54′24″	Beijing Clover Company Beijing, China
*F. arundinacea* ‘road-Fire’	Beijing, China	2022	116°23′51″	39°54′24″	Beijing Clover Company Beijing, China
*F. sinensis*	Ningxia, China	2020	106°28′	36°01′	Lanzhou University Lanzhou, China
*F. sinensis*	Qinghai, China	2020	103°05′	39°15′	Beijing Clover Company Beijing, China
*F. kryloviana*	Qinghai, China	2020	103°05′	39°15′	Lanzhou University Lanzhou, China
*F. rubra* ‘dream-God’	Beijing, China	2022	116°23′51″	39°54′24″	Beijing Clover Company Beijing, China

**Table 2:** Identification of 36 representative bacterial strains from fescue seeds.

Isolate Code	Gram Strain Reaction	Similar Strain	Similarity (%)	Genebank Accession
QY4	−	*Pseudomonas* sp.	100	OR858852
QY2	−	*Pantoea* sp.	100	OR858858
QY5	−	*Pseudomonas* sp.	100	OR858859
QY6	−	*Pantoea* sp.	100	OR858860
LH1	+	*Erwinia* sp.	100	OR858877
LH5	−	*Erwinia* sp.	100	OR858878
LH4	−	*Pantoea* sp.	100	OR858879
LH6	+	*Pseudomonas* sp.	99	OR858880
LH7	+	*Paenibacillus* sp.	100	OR858881
LH8	+	*Bacillus* sp.	100	OR858882
NX2	+	*Bacillus* sp.	100	OR858865
NX6	−	*Bacillus* sp.	100	OR858866
NX3	−	*Bacillus* sp.	100	OR858867
NX5	+	*Pantoea* sp.	86	OR858868
HS8	+	*Paenibacillus* sp.	100	OR858847
HS9	−	*Bacillus* sp.	100	OR858848
HS4	+	*Bacillus* sp.	100	OR858849
HS6	−	*Pseudomonas* sp.	100	OR858850
HS7	+	*Paenibacillus* sp.	95	OR858851
MS8	−	*Curtobacterium* sp.	100	OR858856
MS2	−	*Pantoea* sp.	100	OR858869
MS3	+	*Paenibacillus* sp.	95	OR858870
MS1	+	*Paenibacillus* sp.	100	OR858871
MS4	−	*Paenibacillus* sp.	100	OR858873
MS5	−	*Paenibacillus* sp.	100	OR858872
YY7	−	*Curtobacterium* sp.	100	OR858853
YY13	+	*Chryseobacterium* sp.	100	OR858876
YY15	+	*Curtobacterium* sp.	100	OR858854
YY4	−	*Curtobacterium* sp.	100	OR858855
YY12	+	*Pseudomonas* sp.	91	OR858875
YY11	+	*Exiguobacterium* sp.	100	OR858874
DPX7	−	*Curtobacterium* sp.	100	OR858857
DPX8	−	*Erwinia* sp.	100	OR858861
DPX10	−	*Pseudomonas* sp.	100	OR858862
DPX6	−	*Bacillus* sp.	100	OR858863
DPX9	−	*Stenotrophomonas* sp.	100	OR858864

Note: −: negatives; +: positive.

**Table 3:** Biological function of endophytic bacteria from fescue.

Isolates	IAA (μg/mL; M ± SE)	Nitrogen Fixation	Enzyme	
			Amylase	Protease
QY2	−	+	−	+
QY4	24.13 ± 0.050	+	+	−
QY5	−	+	−	−
QY6	40.10 ± 0.233	+	+	+
LH1	−	−	−	−
LH4	39.48 ± 0.337	−	+	−
LH5	25.44 ± 0.657	−	+	−
LH6	−	−	−	−
LH7	−	−	−	+
LH8	−	−	−	−
NX2	−	+	−	−
NX3	−	+	−	−
NX5	33.49 ± 0.387	+	+	+
NX6	−	+	−	−
HS4	−	−	−	−
HS6	−	+	−	−
HS7	−	+	−	−
HS8	39.11 ± 0.106	+	−	+
HS9	40.88 ± 0.709	+	+	+
MS1	21.57 ± 0.381	+	−	−
MS2	−	+	−	−
MS3	−	+	−	−
MS4	19.53 ± 0.456	+	−	−
MS5	−	+	−	−
MS8	−	−	−	−
YY4	−	+	−	+
YY7	27.38 ± 0.403	−	−	+
YY11	22.63 ± 0.535	+	−	−
YY12	33.88 ± 0.334	+	+	+
YY13	23.46 ± 0.46	+	−	−
YY15	20.48 ± 0.403	+	−	−
DPX6	−	+	−	−
DPX7	34.51 ± 0.414	+	+	+
DPX8	−	−	−	−
DPX9	−	+	−	−
DPX10	−	−	−	+

Note: −: negatives; +: positive.

## Data Availability

Data are contained within the article.
